# Fatigue Performance of Nitrided H13 Tool Steel Pre-Treated with Fine Particle Peening

**DOI:** 10.3390/ma18174121

**Published:** 2025-09-02

**Authors:** Hung-Chi Lee, Tai-Cheng Chen, Wen-Han Chen, Leu-Wen Tsay

**Affiliations:** 1 Department of Optoelectronics and Materials Technology, National Taiwan Ocean University, Keelung 202301, Taiwan; andy82821997@gmail.com; 2 Department of Material Research, National Atomic Research Institute, Taoyuan 325207, Taiwan; tcchen@nari.org.tw; 3 Neutron Scattering Division, Oak Ridge National Laboratory, Oak Ridge, TN 37830, USA; 4 Vincent Vacuum-Tech Co., Ltd., Taoyuan 326019, Taiwan; wenhan@vvt.com.tw

**Keywords:** AISI H13, fine particle peening, gas nitriding, rotating bending fatigue, subsurface crack initiation

## Abstract

This study evaluated the fatigue performance of nitrided H13 steel with and without a compound layer (CL), using two nitrogen potentials (K_N_ = 0.8, designated as LN, and K_N_ = 2.0, designated as HN). Fine particle peening (FPP) was applied prior to gas nitriding to introduce a refined microstructure and compressive residual stress (CRS) in the peened zone. After gas nitriding at 540 °C for 8 h, the refined structure remained on the outermost layer of all samples, regardless of the nitrogen potential. A CL primarily composed of Fe_3_N formed on the external surface of the HN sample, whereas the LN sample remained free of CL. A higher K_N_ promoted CL formation and slightly increased the case depth in the HN sample compared to the LN sample. Fatigue cracks initiated at the external surface of the H13 steel substrate (SB). Overall, the LN and HN samples exhibited similar residual stress fields and, consequently, comparable fatigue performance. In the high-cycle fatigue regime, fatigue cracks originated from subsurface inclusions, resulting in significantly improved fatigue strength and life for both the LN and HN samples compared to the SB sample. Under cyclic stresses at or above 1100 MPa, the crack initiation site in the HN sample tended to shift from subsurface inclusions to the external surface. Throughout the fatigue tests, no multi-cracking or spalling of the CL was observed in the HN sample, regardless of the cyclic stress.

## 1. Introduction

AISI H13 tool steel is widely used for hot-working dies and mandrels due to its combination of high hardenability, fracture toughness, and elevated-temperature strength [1]. The presence of fine alloy carbides within the tempered martensite contributes to the excellent mechanical properties of H13 steel, making it suitable for various hot-working die applications [1,2,3]. Aluminum extrusion is one of the most common processes for producing diverse aluminum profiles, such as beams, tubes, wires, and bars. During extrusion, the die bearing surface is subjected to severe frictional and thermal stresses, abrasion, thermal cycling, and chemical attack [4,5,6]. Moreover, the interaction between the hard oxide film on the aluminum billet and the die surface leads to intense sliding wear [7,8], significantly reducing die service life. Therefore, enhancing the service life of hot-working dies is a critical concern in the aluminum extrusion industry.

Gas nitriding is one of the most widely used thermo-chemical processes for enhancing the surface properties of steel components. It significantly improves abrasion [4,7,8] and fatigue resistance [9,10]. The precipitation of fine chromium nitrides (Cr-nitrides) and nitrogen solution hardening [11] contribute to substantial surface hardening and the introduction of compressive residual stress (CRS) in nitrided steel. In the extrusion industry, gas nitriding of H13 steel is widely employed to improve the wear resistance of aluminum extrusion dies [4,5,6,12,13,14]. The compound layer (CL) formed on the die surface provides greater chemical stability against hot aluminum attack during extrusion compared to untreated surfaces [4,5,6]. Furthermore, nitrided H13 steel exhibits enhanced corrosion resistance in a 3.5% NaCl solution compared to untreated steel [15].

Nitriding is widely used to enhance the fatigue strength and life of steel components [9,10,16,17]. It has been shown to significantly prolong the fatigue life of nitrided steel under low cyclic loading conditions [17]. Fatigue failure of hot-working dies is reported to be one of the primary concerns in the aluminum extrusion industry [18]. In addition to nitriding, shot peening is commonly applied to improve the fatigue resistance of steel components and the wear resistance of hot forging dies [19,20]. Shot peening has also been shown to enhance the thermal fatigue resistance of H13 die-casting molds [21,22]. However, cracks have been observed to initiate within the nitrided layer of H13 mandrel bridges after multiple nitriding treatments, and the susceptibility to cracking increases with greater nitrided layer thickness [23]. Furthermore, the ease of crack initiation in the brittle surface layer of nitrided H13 [18] and D3 steels [24] contributes to the reduction in fatigue strength and life.

Surface preparation prior to nitriding significantly enhances nitriding kinetics, resulting in a more uniform and deeper case depth [25]. Shot peening before nitriding H13 steel increases both the case depth and surface hardness [2,26], attributed to the elevated nitrogen concentration and greater nitrogen diffusion depth in the surface layer [2]. To address the limitations of increased surface roughness for conventional shot peening, fine particle peening (FPP) or micro-shot peening is employed, which decreases surface roughness but induced a relatively narrow compressive stress field in the peened layer [27,28,29,30,31,32,33]. FPP refines the grain structure and introduces CRS into the severely deformed surface zone [28,29,30,31], thereby improving the fatigue performance of treated samples. The enhanced hardness and CRS contribute to the substantial increase in the fatigue limit of nitrided 4140 [16] and 4135 steels pre-treated with FPP [27]. Moreover, applying FPP after carburizing gear steels has been shown to significantly improve scuffing resistance [33].

In this study, FPP was conducted prior to gas nitriding of H13 steel under controlled nitrogen potentials (K_N_). To investigate the influence of nitriding conditions specifically, the presence or absence of a CL on the fatigue performance of nitrided H13 steel, two K_N_ = 2.0 and 0.8 were applied. Rotating bending fatigue tests were performed at room temperature to establish the stress amplitude (S) versus cycle number (N) (S-N) curves of the tested samples. Fracture surfaces of the fatigue-tested samples were examined using a scanning electron microscopy (SEM), and detailed microstructural analyses were conducted via electron backscatter diffraction (EBSD) mapping.

## 2. Material and Experimental Procedures

### 2.1. Preparation of the Samples

**Figure 1:**
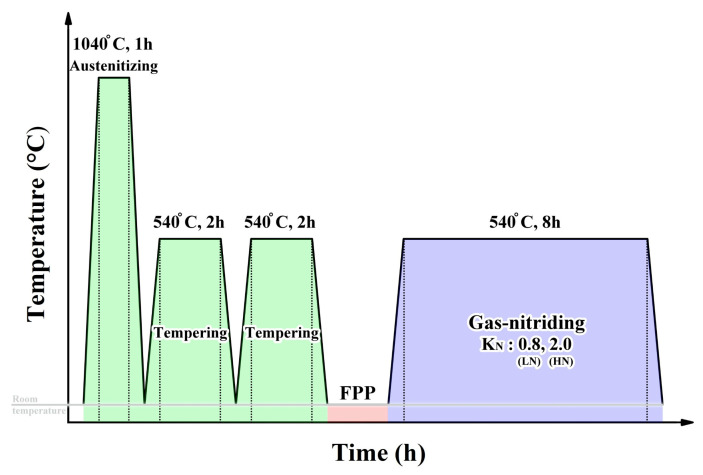
Thermochemical process sequence of the nitriding treatment, with FPP as a pre-treatment.

**Figure 2:**
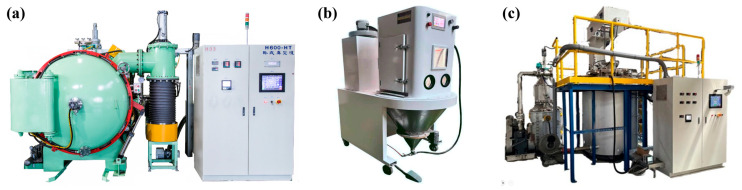
Photographs of the main equipment used in this study: (**a**) austenitizing furnace (Vincent Vacuum-Tech Co., Ltd., Taoyuan, Taiwan), (**b**) FPP setup (Rich Sou Technology Co., Ltd., Kaohsiung, Taiwan), and (**c**) controlled nitrogen-potential furnace (Vincent Vacuum-Tech Co., Ltd., Taoyuan, Taiwan).

Figure 1 illustrates the nitriding process employed in this study, which included FPP prior to nitriding. Figure 2 shows the main equipment used in the experiments. H13 steel bars were first austenitized in an industrial vacuum furnace (Vincent Vacuum-Tech Co., Ltd., Taoyuan, Taiwan) at 1040 °C for 1 h, followed by gas-assisted cooling to room temperature in nitrogen (Figure 2a). Double tempering was then performed at 540 °C for 2 h, with nitrogen gas cooling to room temperature after each cycle. The double-tempered H13 steel was ground with sandpapers ranging from 320 to 1000 grit prior to FPP. The FPP process was performed using amorphous powders as shots, with particle sizes between 80 and 120 μm, under an air pressure of 5 atm. The peening intensity, measured with an N-type Almen specimen, was approximately 0.146 mm in height. Figure 2b shows the facility for FPP conducted at room temperature on the ground H13 steel before gas nitriding. Gas nitriding was conducted in a controlled K_N_ furnace (Figure 2c) using NH_3_ as the nitriding gas and N_2_ as the carrier gas at a pressure of 600 Torr. K_N_ was defined as the ratio of the partial pressures of NH_3_ and H_2_ in the nitriding atmosphere, expressed as K_N_ = P(NH_3_)/P(H_2_)^3/2^. During nitriding, K_N_ values of 0.8 or 2.0 were applied at 540 °C for 8 h, followed by furnace cooling. Based on the applied K_N_, the nitrided samples were designated as LN (K_N_ = 0.8) and HN (K_N_ = 2.0). The un-nitrided H13 steel, which had been austenitized and tempered three times at 540 °C for 2 h, was designated as the substrate (SB).

### 2.2. Measurement of Hardness and Fatigue Testing

The microhardness profile of the nitrided samples was measured at various depths using an MVK-G1500 Vickers hardness tester (Mitutoyo, Kawasaki, Japan). Additionally, a Hysitron TI 980 TriboIndenter (Bruker, Billerica, MA, USA) with a 2000 μN load was used to determine the nanohardness of the CL and/or the diffusion zone (DZ) in the LN and HN samples. The surface topography of the samples was characterized using a Contour GT-K 3D optical profiler (Bruker, Billerica, MA, USA). Rotating bending fatigue tests were performed to assess the effects of FPP and nitriding on the fatigue resistance of H13 steel, in comparison with the untreated substrate (SB) sample. Dog-bone-shaped specimens were used for the rotary bending fatigue tests, which were conducted at a frequency of 1500 cycles per minute in laboratory air. S-N curves were established based on the mean values of three specimens for each testing condition, while individual values were also presented.

### 2.3. Microstructural Observation

A D2 Phaser X-ray diffractometer (XRD, Bruker, Billerica, MA, USA) with Cu Kα radiation was used to identify the phase constituents in the examined specimen, and the results were further confirmed by EBSD analysis. Samples for metallographic examination and microhardness measurement in cross-sectional view were ground with sandpapers up to 1500 grit, followed by polishing with a colloidal silica suspension. The cross-sectional microstructures and fatigue fracture surfaces of the tested samples were examined using an S-3400N SEM (Hitachi, Tokyo, Japan). The chemical composition at various depths from the sample surface was determined by a JXA-8200 electron probe micro-analyzer (EPMA, JEOL, Tokyo, Japan). To further identify distinct phases, the LN and HN samples were analyzed using SEM equipped with a NordlysMax^2^ EBSD detector (Oxford Instruments, Abingdon, UK).

### 2.4. Measurement of Residual Stress

Residual stress measurements were performed using a µ-X360s residual stress analyzer (Pulstec, Hamamatsu, Japan) equipped with Cr Kα radiation, which collected the diffracted beams from the irradiated sample surface. The residual stress was determined using the cos α method [34,35]. The full width at half maximum (FWHM) of the (103) Fe_2_N (2θ = 135.5°) and (211) α (2θ = 156.4°) Debye rings were used to evaluate lattice distortion in the samples. The residual stress was calculated based on the difference in the 2θ angle between the measured Debye rings and the reference phase angles. To obtain the residual stress profile in the thickness direction, successive layers of material were removed using electrochemical polishing.

## 3. Results

### 3.1. Microstructure Identification and EBSD Analysis

**Figure 3:**
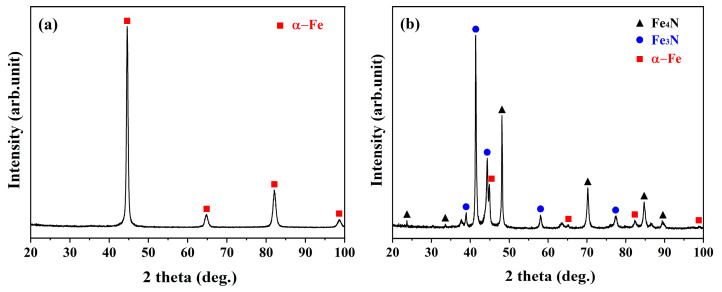
XRD spectra showing the phase constituents detected on the surfaces of the (**a**) LN and (**b**) HN samples.

The phase constituents of the nitrided samples, as identified by XRD, are shown in Figure 3. The XRD pattern revealed that only α-Fe peaks were present in the LN sample (Figure 3a), whereas the HN sample exhibited a predominant mixture of Fe_3_N and Fe_4_N phases on the outer surface (Figure 3b). These results indicated that the low K_N_ used for the LN sample effectively suppressed the formation of a CL on its external surface (Figure 3a).

**Figure 4:**
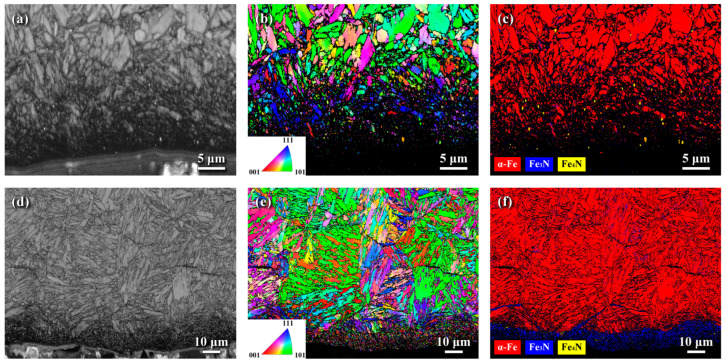
EBSD analysis of the LN and HN samples in cross-sectional view: (**a**,**d**) BC maps; (**b**,**e**) IPF maps; (**c**,**f**) phase maps. (**a**–**c**) correspond to the LN sample; (**d**–**f**) correspond to the HN sample.

Figure 4 presents the EBSD analyses showing the microstructures of the surface zones of the LN and HN samples in cross-sectional view. The band contrast (BC) maps (Figure 4a,d) illustrate the overall microstructure; the inverse pole figure (IPF) maps (Figure 4b,e) display grain orientations indicated by distinct colors; and the phase maps (Figure 4c,f) identify the phase constituents in the examined regions. The BC maps (Figure 4a,d) reveal a thin dark zone near the top surface and show lath martensitic packets with varying orientations in both the LN and HN samples. Previous studies have indicated that FPP promotes the formation of nanograins in the outermost surface of shot-peened steel [16]. Consistent with this, extremely fine grains were observed in the outermost zones of both the LN and HN samples (Figure 4b,e). Due to the limited resolution of EBSD analysis, the surface grain sizes of both samples were too fine to be distinctly resolved, appearing instead as a vaguely dark layer near the top surface. However, a few micrometers beneath the surface, refined grains were observed above the underlying regular structure. Figure 4e displays that the grain refinement induced by FPP was retained after gas nitriding at 540 °C. The phase map of the LN sample (Figure 4c) confirmed the absence of a CL, consistent with the XRD results (Figure 3a). In contrast, a CL primarily composed of Fe_2–3_N, with a thickness less than 10 μm, was observed in the surface zone of the HN sample (Figure 4f). The CL thickness fluctuated between 6 and 9 μm in the HN sample. The phase map (Figure 4f) revealed that dense Fe-nitride precipitates decorated the lath martensite and austenite grain boundaries in the HN sample. This finding indicates that nitrogen atoms preferentially diffused along the refined grains and austenite grain boundaries of the nitrided H13 steel. These results further suggest that the refined granular structure produced by FPP promoted nitrogen penetration into the interior, thereby providing additional diffusion paths that facilitated the formation of nitrided phases in the surface zone and increased the case depth of the steel.

### 3.2. Surface Texture of the Nitrided Samples

**Figure 5:**
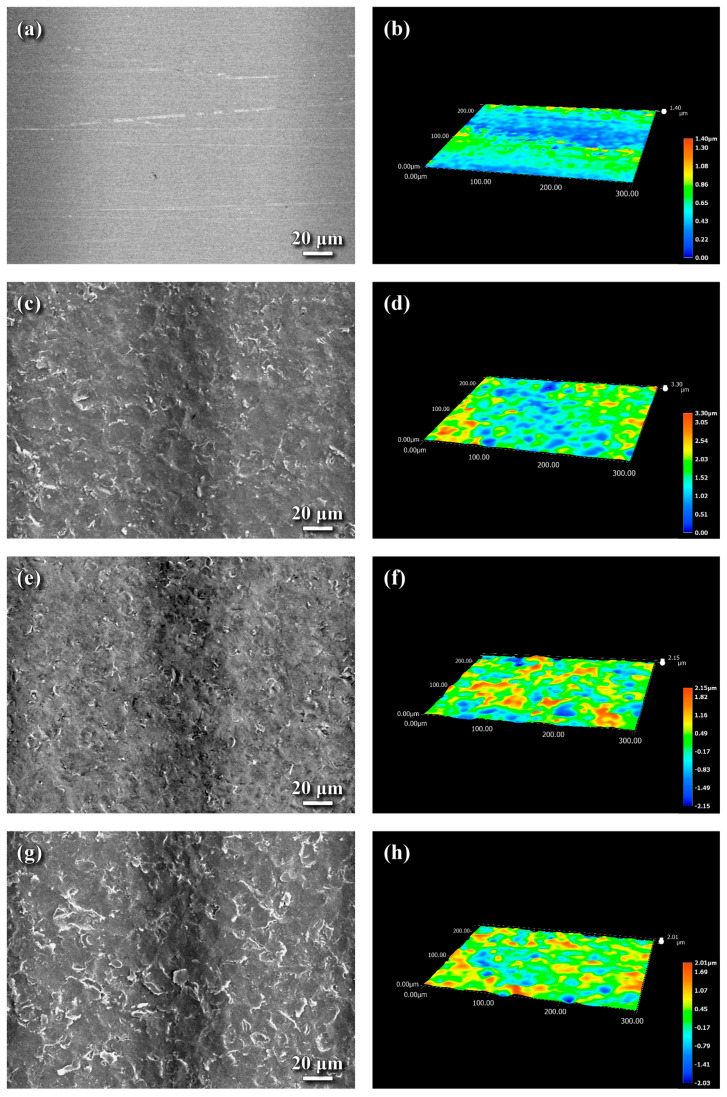
SEM surface morphology (**a**,**c**,**e**,**g**) and 3D surface topography (**b**,**d**,**f**,**h**) of the (**a**,**b**) SB, (**c**,**d**) SP, (**e**,**f**) LN, and (**g**,**h**) HN specimens.

Figure 5 presents the surface morphology and topography of the SB, SP, LN, and HN samples, as analyzed by an optical profiler. The measured surface roughness values for the investigated samples are summarized in Table 1. The substrate (SB sample), prepared by grinding with 1000-grit sandpaper, exhibited visible grinding marks in the SEM images (Figure 5a). The surface roughness values of the SB sample were Sa = 0.14 μm, Sp = 0.86 μm, and Sv = 0.55 μm. After FPP treatment, the surface roughness of the shot-peened (SP) sample increased to Sa = 0.39 μm, Sp = 1.70 μm, and Sv = 1.60 μm, indicating that FPP substantially increased surface roughness compared to the ground SB sample. This difference is displayed in the surface topographies of the SB and SP samples (Figure 5b,d). The LN sample exhibited surface roughness values of Sa = 0.45 μm, Sp = 1.75 μm, and Sv = 2.29 μm. The similarity in roughness between the SP and LN samples suggests that FPP was the primary contributor to the increased surface roughness of the LN sample compared to the SB sample. The surface topography of the LN and HN samples is shown in Figure 5f and Figure 5h, respectively. As listed in Table 1, the HN sample exhibited slightly higher surface roughness values (Sa = 0.55 μm, Sp = 1.94 μm, Sv = 2.36 μm) compared to the LN sample. Both the LN and HN samples showed similar surface features (Figure 5e,g) characterized by peened dents remaining on the top surface. However, the HN sample displayed a distinct texture with interspersed fine dents and debris on its surface (Figure 5g).

### 3.3. Microstructure of the Tested Samples

**Figure 6:**
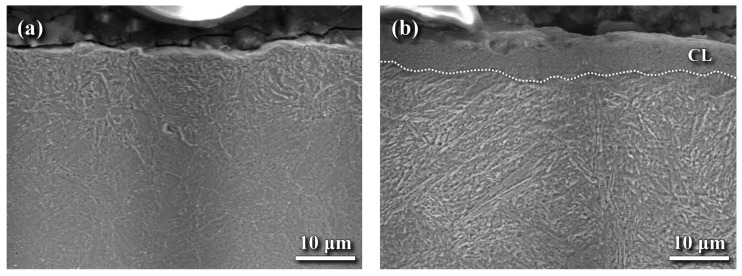
SEM cross-sectional microstructures near the outer surfaces of the (**a**) LN and (**b**) HN samples. The dash line in (**b**) indicates the boundary of the CL.

Figure 6 presents the SEM cross-sectional microstructures near the outer surfaces of the LN and HN samples. No CLwas observed on the LN sample (Figure 6a), whereas the HN sample exhibited a thin CL on its top surface (Figure 6b). The CL in the HN sample contained shallow surface defects, which may be detrimental to its fatigue resistance. Beneath the surface, the DZ and substrate in both samples displayed a lath-like tempered martensite dispersed with fine precipitates. Table 2 summarizes the chemical compositions measured by EPMA at various depths from the top surface of the HN sample. The results show that the nitrogen (N) content in the surface zone of the CL exceeded 4 wt%. Within the CL, the nitrogen content gradually decreased while the carbon (C) content increased with increasing depth from the surface. The concentrations of other alloying elements remained relatively constant within the CL. In the DZ, a significant decrease in nitrogen content was observed, dropping from approximately 1.61 wt% at a depth of 15 μm to 1.05 wt% at 45 μm. Beyond a depth of 75 μm, the nitrogen content reduced to a negligible level. These findings suggested that high nitrogen concentration near the outermost surface of the HN sample corresponded to the formation of the CL. The low oxygen (O) concentration near the surface indicated that oxygen contamination during gas nitriding was negligible.

### 3.4. Hardness Profile of the Nitrided Samples

**Figure 7:**
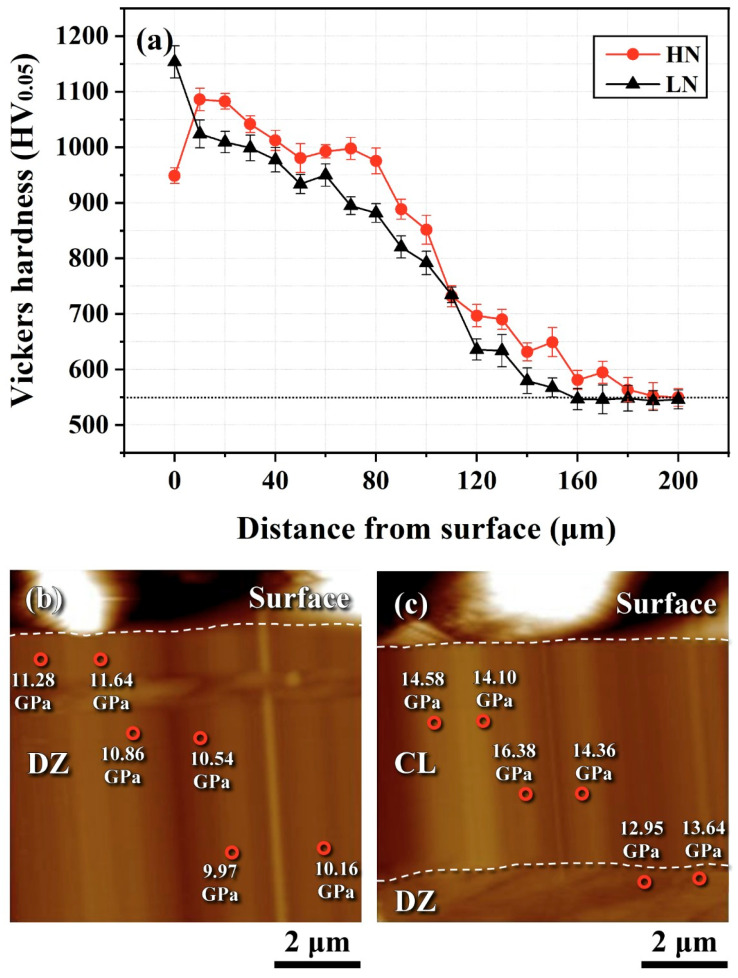
(**a**) Micro-Vickers hardness profiles of the LN and HN samples from the outermost surface to the core; (**b**) nanohardness of the DZ in the LN sample; (**c**) nanohardness of the CL and DZ in the HN sample.

Figure 7 shows the microhardness and nanohardness distributions from the external surface to the core of the LN and HN samples. The case depth was defined as the distance from the outermost surface to the location where the hardness was HV 50 higher than the core hardness (approximately HV 550). The surface layer of the nitrided H13 steel exhibited significantly higher hardness than the core (Figure 7a), regardless of the nitriding condition. The hardness profiles of both nitrided samples exhibited a sharp decrease from approximately HV 1000 at the surface to HV 550 in the interior, with the transition occurring at a depth of ~160 μm for the LN sample and ~200 μm for the HN sample. The presence of a hardened case in both samples indicates sufficient nitrogen diffusion during the nitriding process. The HN sample had a slightly greater case depth than the LN sample. Surface defects present in the CL led to a reduction in the superficial microhardness of the HN sample (Figure 7a). Figure 7b and Figure 7c display the nanohardness of the DZ and CL for the LN and HN samples, respectively. The DZ of the LN sample exhibited a nanohardness of approximately 10 GPa (equivalent to HV 1040) within the measured region (Figure 7b). In contrast, the CL of the HN sample reached a nanohardness as high as 16.38 GPa (Figure 7c). The DZ beneath the CL of the HN sample had a nanohardness of about 13 GPa (HV 1300). These results demonstrate that the CL in the HN sample was harder than the underlying DZ. Comparing the DZ hardness of the LN and HN samples, the HN sample exhibited a significantly higher hardness. This difference may partially result from the reduced nitrogen diffusion and concentration in the LN sample nitrided at the lower K_N_.

### 3.5. Residual Stress Measurements

**Figure 8:**
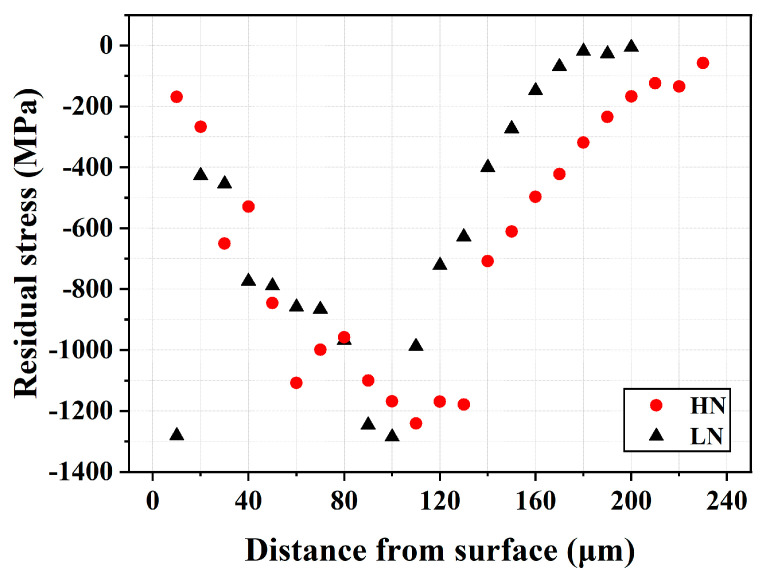
Residual stress profiles along with the thickness direction of the LN and HN samples.

Figure 8 presents the residual stress profiles along the thickness direction of the LN and HN samples. Due to the irregular top surface, the HN sample exhibited greater variation in the measured residual stress near the surface. The results indicated that the combined effects of gas nitriding and FPP introduced high CRS into the treated material. Overall, the LN and HN samples displayed similar residual stress profiles. The maximum compressive stress was observed in the subsurface region of both specimens. The CRS reached a maximum of approximately 1300 MPa at a depth of around 100 μm from the external surface in both samples. In addition, the CRS field in the HN sample extended slightly deeper into the interior of the sample than that of the LN one. Given the limited difference in CRS field between the LN and HN samples, it is likely that both samples would exhibit comparable fatigue properties. As reported in previous studies [16,27,28,29,30], FPP or micro-shot peening can introduce high CRS into the severely peened zone, but the affected depth is relatively shallow. High CRS is expected to exert a significant effect in enhancing the fatigue resistance of the peened material.

### 3.6. Fatigue Tests

**Figure 9:**
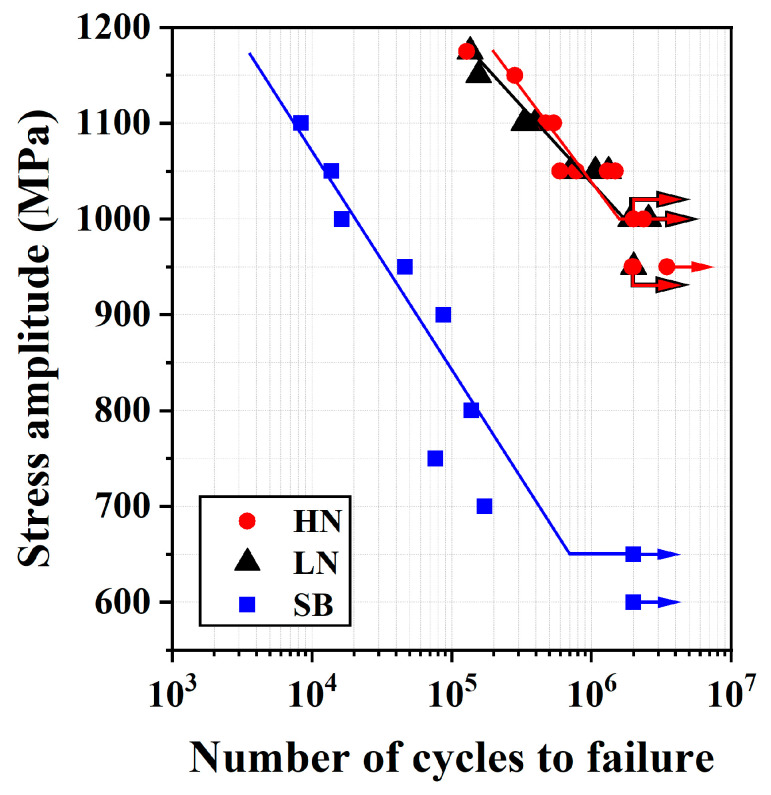
The S-N curves for the LN and HN samples, compared with the SB sample, tempered three times at 540 °C for 2 h.

The S-N curves obtained from the rotating bending fatigue tests are shown in Figure 9. After multiple tempering treatments at 540 °C, the H13 steel retained a high core hardness of approximately HV 550. The fatigue test results revealed a sharp decrease in the fatigue life of the SB sample with increasing cyclic stress. The fatigue limit of the SB sample was approximately 650 MPa. In contrast, the LN and HN samples exhibited similar S-N curves, with fatigue limits reaching around 1000 MPa, significantly higher than that of the SB sample. These results demonstrated that the combination of FPP and gas nitriding markedly improved the fatigue strength/life of H13 steel. Although the LN and HN samples possessed different superficial microstructures, they exhibited nearly identical fatigue performance. This suggests that the brittle CL, which is typically beneficial for enhancing wear resistance, did not strongly damage the fatigue performance of the nitrided H13 steel.

### 3.7. Fractured Surface Examinations

**Figure 10:**
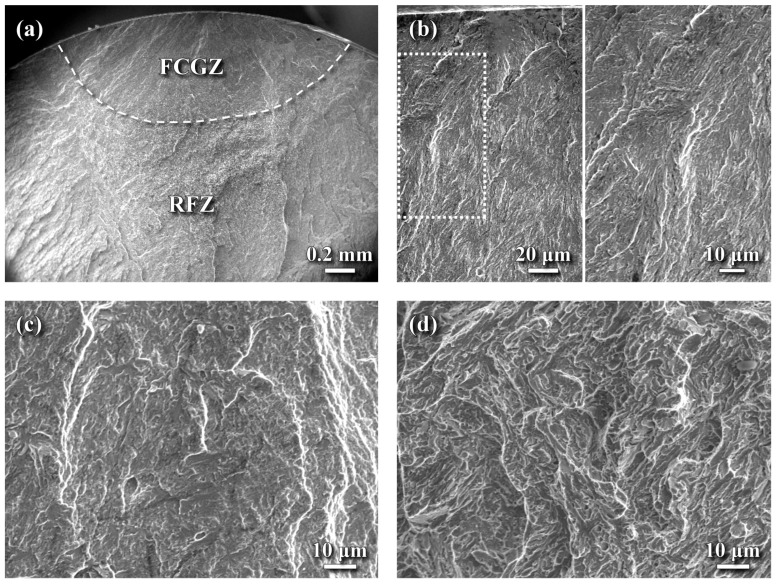
SEM fatigue fracture features of the SB sample: (**a**) macro-fractured appearance, (**b**) transgranular brittle fracture at the crack initiation site, (**c**) quasi-cleavage fracture with tearing ridges in the FCGZ, and (**d**) quasi-cleavage with fine, elongated facets in the RFZ.

The fatigue fracture morphology of the SB sample is shown in Figure 10. During the fatigue test, the peak tensile stress generated on the outer surface of the specimen typically initiated cracks at the surface, which then propagated inward. Figure 10a–b demonstrate that fatigue cracks tend to initiate at the external surface of the SB sample and extend toward the interior. In the crack initiation zone (Figure 10b), a quasi-cleavage fracture featuring traces of the lath texture was observed. Within the fatigue crack growth zone (FCGZ), at greater depths from the surface, an increase in crack growth rate was accompanied by the appearance of tearing ridges (Figure 10c). Parallel elongated facets observed in this region were likely associated with the lath packets of the tempered martensite. It was observed that a transition in fracture morphology (Figure 10a) was evident from a relatively smooth surface near the crack origin to a coarser texture outside the FCGZ. The rapid fracture zone (RFZ) displayed a quasi-cleavage fracture characterized by rough surface with fine and elongated facets (Figure 10d).

**Figure 11:**
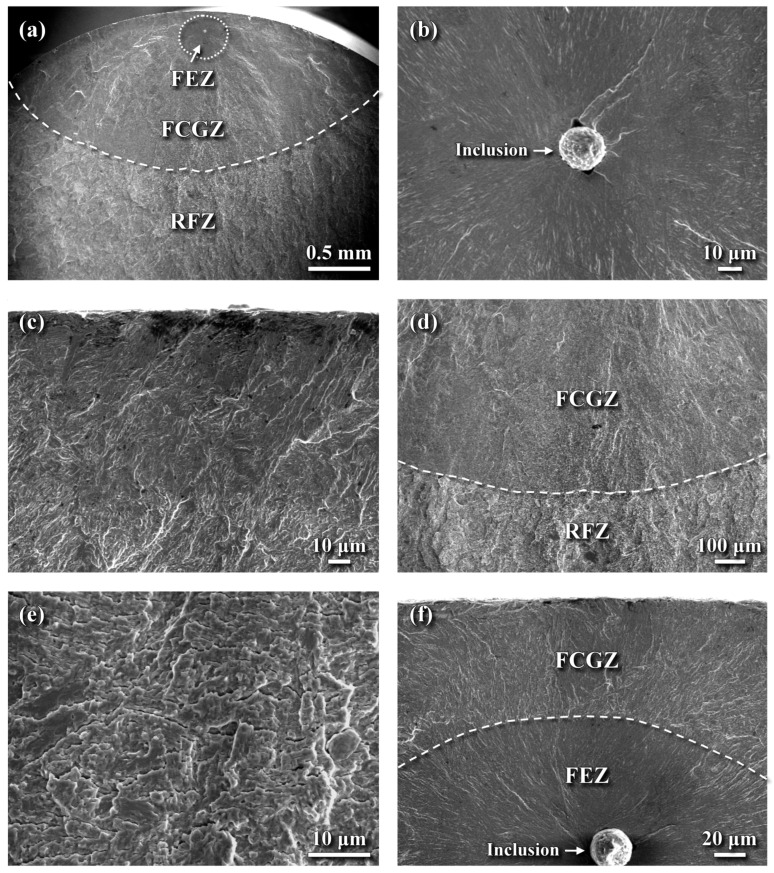
SEM fracture morphology of the LN sample: (**a**) macro-fractured appearance, (**b**) crack initiation from a subsurface inclusion, (**c**) fracture features of the outermost surface, (**d**) transition in appearance from the FCGZ to the RFZ, (**e**) quasi-cleavage fracture interspersed with microcracks in the FCGZ, and (**f**) subsurface crack initiation under a fatigue stress of 1150 MPa.

The fatigue fracture morphology of the LN sample is shown in Figure 11. In the high-cycle fatigue regime, a subsurface fish-eye zone (FEZ) was observed (Figure 11a), indicating that fatigue crack initiation and early growth occurred from this region. The macro-fracture surface of the LN sample (Figure 11a) also revealed a faint dark layer along the outer profile of the fracture surface. The fatigue crack propagated more rapidly inward than outward toward the external surface, leading to final rupture. The FEZ exhibited a smooth, cleavage-like fracture with radial markings emanating from a subsurface inclusion (Figure 11b). EPMA analysis identified the chemical composition of this inclusion (in wt%) as 15.18 C, 39.02 O, 4.76 Al, 22.79 Ca, 2.13 Si, 0.51 Mn, with the balance being Fe. Based on this composition, the inclusion was deduced to be an Al-Ca oxide, with carbon co-segregated within it. A thin dark layer approximately 100 μm thick was present on the outer profile of the fractured LN sample (Figure 11a). Examination of the outer surface layer (Figure 11c) revealed a rubbed surface texture with visible lath martensitic morphology. Figure 11d illustrates the transition in fracture appearance from the FCGZ to the RFZ. Within the FCGZ, fine facet fractures interspersed with numerous microcracks were observed (Figure 11e). In contrast, the RFZ exhibited a quasi-cleavage fracture interspersed with fine facets of various sizes and shapes, which was similar to the fracture appearance in the SB sample (Figure 10d). It was noted that subsurface crack initiation, characterized by the formation of an FEZ, was more likely to occur in the LN sample, even under peak cyclic stress of 1150 MPa (Figure 11f). The shortened fatigue life observed at this stress level was associated with a smaller FEZ and shorter crack growth path toward the interior of the LN sample (Figure 11f).

**Figure 12:**
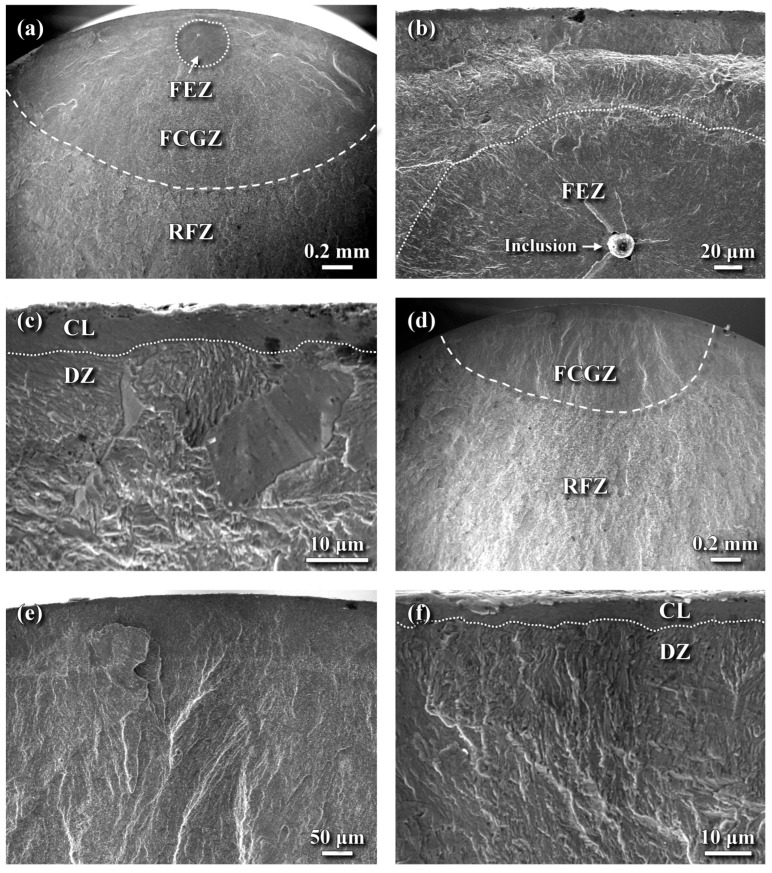
SEM fracture morphology of the HN sample: (**a**) macro-fractured appearance showing subsurface crack initiation, (**b**) deflected crack path between the FEZ and the top surface, (**c**) enlarged view of the CL and DZ in the high-cycle fatigue regime, (**d**) surface crack initiation at peak stresses at or above 1100 MPa, (**e**) enlarged view of the surface morphology shown in (**d**), and (**f**) enlarged view of the CL and the underlying DZ.

Figure 12 shows the fatigue fracture morphology of the HN sample. Similar to the LN sample, fatigue crack initiation in the HN sample occurred from a subsurface FEZ in the high-cycle fatigue regime (Figure 12a). Subsurface fatigue crack initiation is strongly influenced by the quantity and distribution of inclusions in the material. Like the LN sample, a thin dark layer approximately 100 μm thick was observed along the outer periphery of the fractured HN sample. Examining the near-surface fracture morphology (Figure 12b–c) revealed a deflected crack path between the FEZ and the outermost layer (Figure 12b). Although the CL is inherently brittle, no evidence of cracking or spalling was observed (Figure 12c). Beneath the CL, the DZ (Figure 12c) exhibited a relatively rough surface and a lath martensitic texture. As the crack propagated inward from the FEZ, the HN sample displayed fracture morphologies similar to those of the LN sample in the corresponding regions (Figure 11d–e). Notably, at peak cyclic stresses at or above 1100 MPa, crack initiation shifted from the subsurface region to the outer surface of the HN sample (Figure 12d). Even under such high stresses, a rubbed layer approximately 60 μm thick was still visible along the outer profile of the HN sample (Figure 12e). High-magnification observations of the outermost zone (Figure 12f) showed that the CL remained intact and continuous. Beneath the CL, fatigue cracks tended to propagate directly into the interior, displaying a lamellar texture normal to the external surface. In contrast, in the high-cycle fatigue regime, the lamellar texture beneath the CL exhibited multiple orientations (Figure 12c). The shift in the crack initiation site from subsurface to surface at higher fatigue stress in the HN sample might be partially owing to the presence of fine dents on its top surface to induce surface microcracks (Figure 12f).

## 4. Discussion

Table 1 summarizes the surface roughness measurements of the examined samples. The substrate (SB sample) in its ground condition exhibited the lowest surface roughness, while the HN sample showed the highest. However, the difference in surface roughness between the LN and HN samples was relatively small, indicating that the nitriding conditions (specifically, the K_N_) did not significantly influence the surface roughness of the treated samples. In contrast, when comparing the surface roughness of the SB, SP, and LN samples, FPP played a major role in increasing the surface roughness of H13 steel. These results suggest that gas nitriding following FPP contributed only a minor additional increase in surface roughness. The higher surface roughness of the HN sample, compared to the other samples, may be partially owing to the presence of interspersed fine dents and surface debris on the CL.

Conventional gas nitriding is typically carried out at 500–600 °C for up to 100 h. Pre-nitriding shot peening has been reported to increase the case depth and shorten the required nitriding time [2,25,26]. In the peened layer, defects such as dislocation cells, dislocation walls, and mechanical twins provide additional diffusion paths for nitrogen, thereby promoting the formation of a thicker and harder nitrided layer [36]. In this work, FPPwas applied prior to gas nitriding to introduce a refined microstructure and high CRS in the peened zone. A CL was formed on the HN sample (Figure 6b), whereas the LN sample remained free of a CL (Figure 6a). Under the same nitriding duration, the case depth of the LN sample was slightly shallower than that of the HN sample (Figure 7a). Nanohardness measurements confirmed that the CL in the HN sample was harder than the DZ beneath it, indicating that Fe-nitride is harder than the nitrogen-enriched martensite. Compared to the DZ nanohardness of the LN and HN sample (Figure 7b–c), the HN sample exhibited a higher hardness (~13 GPa) than the LN sample (~11 GPa). This difference was attributed to the lower nitrogen supply under the low K_N_ condition of the latter. In addition, the presence of the CL in the HN sample is expected to enhance wear resistance compared to the LN sample without a CL.

EPMA analysis revealed that the nitrogen content exceeded 4 wt% and was primarily concentrated in the outermost CL of the HN sample. A sharp decrease in nitrogen content was observed with increasing depth toward the core of the H13 steel. Even in the DZ directly beneath the CL, the nitrogen content was significantly lower than that in the CL. Similar results have been reported for QPQ-treated 4140 steel [37], where the CL exhibited much higher nitrogen content compared to the underlying DZ. At a depth of 75 μm from the top surface, the nitrogen content decreased to a negligible level. However, the measured hardness at this depth (~HV 1000) remained much higher than that of the substrate (HV 550). This suggests that the nitrogen content determined by EPMA in the DZ was less sensitive in reflecting the hardening effect compared to the hardness measurements.

The size of inclusions and their depth beneath the surface play a critical role in determining the rotating bending fatigue life of bearing steels, often having a greater influence than the inclusion type itself [38]. Fatigue life generally decreases as the inclusion size increases and the depth to the surface decreases [38]. For example, in 35CrMo steel subjected to nitrocarburizing and post-oxidizing treatments, fatigue cracks initiate from subsurface regions in the short fatigue life regime, while cracks originate from internal inclusions during the very high-cycle fatigue regime [39]. In medium-carbon steel gas-nitrocarburized at 570 °C, rotating bending fatigue tests were conducted on specimens in three conditions: as-nitrided, oxide-removed, and oxide + CL-removed states [40]. Interestingly, all these samples exhibited similar fatigue limits regardless of surface preparation [40]. After removing the oxide layer, fatigue fractures initiated from the CL in the short fatigue life regime but shifted to subsurface regions in the very high-cycle fatigue regime [40]. When both the oxide layer and the CL were removed, fatigue cracks initiated from the surface in the short-life regime and from matrix facets during very high-cycle fatigue [40]. In the case of QPQ-treated 4140 steel, cracking and spalling of the superficial CL led to fatigue crack initiation under loading conditions at or above 875 MPa. At slightly lower stresses, near the fatigue limit, crack initiation shifted to subsurface inclusions [37].

In this study, the LN and HN samples exhibited similar fatigue performance, likely due in part to their comparable CRS fields. In the high-cycle fatigue regime, subsurface cracks initiated at inclusions and then propagated both outward and inward before final fracture in both the LN and HN samples. The FEZ in both samples displayed a smooth, cleavage-like fracture with radial traces originating from the inclusion. In the HN sample, a deflected crack path was observed between the FEZ and the outermost surface layer (Figure 12b). Compared to the FEZ, the DZ beneath the CL exhibited a relatively rough fracture surface and lath martensitic texture (Figure 12c). Notably, no cracking or spalling of the CL was observed under any of the applied loading conditions. At higher fatigue stress, fine dents formed on the HN surface were more likely to shift the crack initiation site from the subsurface to the top surface. The CL, which exhibited a rubbed surface appearance rather than cleavage-like fracture, was attributed to the act of CRS to promote crack closure. Thus, the combined effects of CRS and the hardened case acted synergistically to suppress slip activity on the external surface.

FPP prior to gas nitriding was confirmed to be an effective approach for retarding fatigue crack initiation and growth at the surface of nitrided H13 steel. As noted in this work, the influence of the CLon the fatigue performance of nitrided steel remains controversial [23,24,37,38,39,40]. In the HN sample, the surficial defects of the CL could promote surface crack initiation and reduce fatigue life under high cyclic stress. Accordingly, reducing the surficial defects of the CL by polishing or SMAP (Shot Machine A One Polish) treatment [41] is expected to enhance reliability and improve the fatigue performance of FPP + nitrided steel.

## 5. Conclusions

1. Fine particle peening was performed prior to gas nitriding under two nitrogen potentials: K_N_ = 0.8 (designated as LN) and K_N_ = 2.0 (designated as HN). Among the tested samples, the substrate (SB) sample exhibited the lowest surface roughness, while the HN sample showed the highest. Gas nitriding following fine particle peening resulted in only a slight increase in surface roughness. Reducing the nitriding potential (K_N_) from 2.0 to 0.8 suppressed the formation of the compound layer and led to a slight reduction in case depth. The compound layer in the HN sample was primarily composed of Fe_3_N with some Fe_4_N. Phase mapping indicated that nitrogen atoms preferentially diffused along refined grain boundaries and austenite grain boundaries.
2. Nanohardness measurements confirmed that the compound layer in the HN sample was harder than the underlying diffusion zone. EPMA analysis revealed a significant difference in nitrogen content between the compound layer and the diffusion zone. The outermost compound layer contained more than 4 wt% nitrogen and the diffusion zone contained approximately 1.6 wt%. Comparing the nanohardness of the LN and HN samples in diffusion zone, the HN sample (~13 GPa) was clearly harder than the LN sample (~11 GPa). This difference may be partly resulted from the reduced nitrogen diffusion into the H13 steel under the lower K_N_.
3. Both the LN and HN samples demonstrated superior fatigue strength and life compared to the SB sample. The synergistic effect of the compressive residual stress and the hardened case inhibited the occurrence of slips at the external surface, thereby delaying the initiation and propagation of superficial fatigue cracks in the nitrided H13 steel pre-treated by fine particle peening. Similar fatigue performance of the LN and HN samples was likely due to their comparable compressive residual stress fields.
4. Subsurface fatigue crack initiation in the LN and HN samples was associated with the formation of a fish-eye zone, characterized by a smooth, cleavage-like fracture with radial traces originating from an inclusion. In the HN sample, a deflected crack path was observed between the fish-eye zone and the outermost layer. Compared to the fish-eye zone, the diffusion zone beneath the compound layer in the HN sample exhibited a relatively rough fracture surface and lath martensitic texture. Notably, no cracking or spalling of the compound layer occurred under distinct loading conditions in this work. It was suggested that the compound layer, exhibiting a rubbed surface rather than a cleavage-like fracture, was subjected to compressive residual stress, promoting crack closure. With respect to the crack initiation site, the defective CL on the top surface of the HN sample was detrimental to its fatigue resistance, whereas no such feature was found in the LN sample.

## Figures and Tables

**Table 1:** The surface roughness values of the tested samples.

Sample	Surface Roughness (μm)		
	Sa ^1^	Sp ^2^	Sv ^3^
SB	0.14	0.86	0.55
SP	0.39	1.70	1.60
LN	0.45	1.75	2.29
HN	0.55	1.94	2.36

^1^ Sa—arithmetical mean height of the surface. ^2^ Sp—maximum peak height of the surface. ^3^ Sv—maximum pit depth of the surface.

**Table 2:** The chemical compositions measured by EPMA at various depths from the top surface of the HN sample.

Location	Distance from Surface (μm)	Chemical Composition in wt%								
		C	N	O	Mn	Si	Cr	Mo	V	Fe
Compound layer	2	0.23	4.14	0.03	0.34	0.94	5.02	1.19	0.67	Bal.
	4	0.33	4.19	0.02	0.38	0.96	4.82	1.04	0.57	Bal.
	6	0.40	3.66	0.02	0.37	0.93	4.85	1.02	0.56	Bal.
Diffusion zone	15	0.44	1.61	0.07	0.43	1.05	5.85	1.24	0.74	Bal.
	45	0.46	1.05	0.04	0.40	0.95	5.66	1.15	0.71	Bal.
	75	0.35	**-**	0.03	0.39	0.91	5.33	1.16	0.66	Bal.
	140	0.38	**-**	0.01	0.41	0.95	5.56	1.10	0.76	Bal.
Substrate	200	0.35	-	0.01	0.47	0.93	5.62	1.13	0.71	Bal.

## Data Availability

The original contributions presented in this study are included in the article. Further inquiries can be directed to the corresponding author.
